# Electrochemotherapy for the treatment of cutaneous squamous cell carcinoma: The INSPECT experience (2008-2020)

**DOI:** 10.3389/fonc.2022.951662

**Published:** 2022-09-20

**Authors:** Giulia Bertino, Ales Groselj, Luca G. Campana, Christian Kunte, Hadrian Schepler, Julie Gehl, Tobian Muir, James A. P. Clover, Pietro Quaglino, Erika Kis, Matteo Mascherini, Brian Bisase, Giancarlo Pecorari, Falk Bechara, Paolo Matteucci, Joy Odili, Francesco Russano, Antonio Orlando, Rowan Pritchard-Jones, Graeme Moir, David Mowatt, Barbara Silvestri, Veronica Seccia, Werner Saxinger, Francesca de Terlizzi, Gregor Sersa

**Affiliations:** ^1^ Department of Otolaryngology Head Neck Surgery, University of Pavia, Istituto di Ricovero e Cura a Carattere Scientifico (IRCCS) Policlinico San Matteo Foundation, Pavia, Italy; ^2^ Department of Otorhinolaryngology and Cervicofacial Surgery, University Medical Centre Ljubljana, Ljubljana, Slovenia; ^3^ Department of Surgery, The Christie National Health Service (NHS) Foundation Trust, Manchester, United Kingdom; ^4^ Department of Dermatosurgery and Dermatology, Artemed Fachklinik Munich, München, Germany; ^5^ Department of Dermatology, University Medical Center, Johannes Gutenberg University, Mainz, Germany; ^6^ Center for Experimental Drug and Gene Electrotransfer (CEDGE), Department of Clinical Oncology and Palliative Care, Zealand University Hospital, Roskilde, Denmark; ^7^ Department of Clinical Medicine, Faculty of Health and Medical Sciences, University of Copenhagen, Copenhagen, Denmark; ^8^ Department of Reconstructive Plastic Surgery, James Cook University Hospital, Middlesbrough, United Kingdom; ^9^ Department of Plastic Surgery, Cork University Hospital, Cork, Ireland; ^10^ Cancer Research @UCC, University College Cork, Cork, Ireland; ^11^ Dermatologic Clinic, Department of Medical Sciences, University of Turin, Turin, Italy; ^12^ Department of Dermatology and Allergology, University of Szeged, Szeged, Hungary; ^13^ Department of Surgical Sciences, Polyclinic Hospital San Martino, Genoa, Italy; ^14^ Clinic for Head and Neck Cancer, Queen Victoria Hospital National Health Service (NHS) Foundation Trust, East Grinstead, United Kingdom; ^15^ Department of Surgical Sciences, Otolaryngology Clinic, University of Turin, Turin, Italy; ^16^ Department of Dermatologic Surgery, Ruhr-University Bochum, Bochum, Germany; ^17^ Dept Plastic Surgery, Hull University Teaching Hospitals National Health Service (NHS) Trust, Hull, United Kingdom; ^18^ Department of Plastic Surgery, St. Georges University Hospitals National Health Service (NHS) Trust, London, United Kingdom; ^19^ Melanoma and Sarcoma Surgical Oncology Unit, Veneto Institute of Oncology Istituto di Ricovero e Cura a Carattere Scientifico (IRCCS), Padova, Italy; ^20^ Southmead Hospital, North Bristol National Health Service (NHS) Trust, Department of Plastic and Reconstructive Surgery, Bristol, United Kingdom; ^21^ Plastic Reconstructive Surgery, University of Liverpool, St. Helens & Knowsley Teaching Hospitals National Health Service (NHS) Trust, Liverpool, United Kingdom; ^22^ Department of Cutaneous Medicine and Surgery, The Royal London Hospital & Queen Mary University of London (QMUL), Bart’s Health National Health Service (NHS) Trust, London, United Kingdom; ^23^ Department of Surgery, Christie Hospital, Manchester, United Kingdom; ^24^ Oncology and Haematology Unit, Azienda Unità Sanitaria Locale Socio Sanitaria (AULSS) 3 Serenissima-Mirano, Venice, Italy; ^25^ Otorhinolaryngology Audiology and Phoniatric Unit, University Hospital of Pisa, Pisa, Italy; ^26^ Department of Dermatology, Klinikum Wels-Grieskirchen, Wels, Austria; ^27^ Biophysics Lab., IGEA S.p.a, Carpi, Italy; ^28^ Department of Experimental Oncology, Institute of Oncology Ljubljana, Ljubljana, Slovenia; ^29^ Faculty of Health Sciences, University of Ljubljana, Ljubljana, Slovenia

**Keywords:** cutaneous squamous cell carcinoma, electrochemotherapy, skin cancer, local treatment, inspect

## Abstract

**Introduction:**

Cutaneous squamous cell carcinoma (cSCC) is a frequent skin cancer with a high risk of recurrence characterized by tumor infiltration and, in advanced cases, a poor prognosis. ECT (electrochemotherapy) is an alternative treatment option for locally advanced or recurrent cSCC that is unsuitable for surgical resection. In this study, we aimed to evaluate the data in the InspECT (International Network for Sharing Practice on ECT) registry of the referral centers and to clarify the indications for the use of ECT as a treatment modality for cSCC.

**Materials and methods:**

Patients with primary, recurrent or locally advanced cSCC from 18 European centers were included. They underwent at least one ECT session with bleomycin between February 2008 and November 2020, which was performed following the European Standard Operating Procedures.

**Results:**

The analysis included 162 patients (mean age of 80 years; median, 1 lesion/patient). Side effects were mainly local and mild (hyperpigmentation, 11%; ulceration, 11%; suppuration, 4%). The response to treatment per patient was 62% complete and 21% partial. In the multivariate model, intravenous drug administration and small tumor size showed a significant association with a positive outcome (objective response). One-year local progression-free survival was significantly better (p<0.001) in patients with primary tumors (80% (95% C.I. 70%-90%) than in patients with locally advanced disease (49% (95% C.I. 30%-68%).

**Conclusion:**

In the present study, ECT showed antitumor activity and a favorable safety profile in patients with complex cSCC for whom there was no widely accepted standard of care. Better results were obtained in primary and small tumors (<3 cm) using intravenous bleomycin administration.

## Introduction

Cutaneous squamous cell carcinoma (cSCC) represents the second most frequent skin cancer (20%), after BCC, with an incidence of approximately 28.9 per 100 000 inhabitants/year (1). In 1-5% of cases, it can give distant metastases, and such metastases are associated with an average survival of two years (2). Molecularly, cSCC arises from the accumulation of genetic and epigenetic alterations in keratinocytes over time that permit development of an invasive tumor. Frequently it is associated with mutations of tyrosin kinase receptors, certain cell cycle regulatory genes or RAS/MAPK and PI3K signalling pathways. These genetic alterations drive the development of malignant and premalignant lesions, that is accelerated when intrinsic defenses are compromised. The accumulation of genetic alterations, and development of malignant and premalignant lesions, is accelerated when intrinsic defenses are compromised. Examples include patients on chronic immunosuppression, or those with a heritable predisposition to cancer, such as the disease states of Xeroderma Pigmentosum or Bloom syndrome (3, 4).

The primary goal of cSCC therapy is to completely remove the tumor with maximal functional and cosmetic preservation. Surgical excision alone guarantees the successful treatment of cSCC, with a good prognosis and cure rates greater than 90% (5).

In patients not amenable to surgery, radiotherapy, eventually associated with chemotherapy, represents a valid and curative treatment strategy for cSCC (5). Local recurrences of cSCC frequently exhibit a more extensive, irregular subclinical infiltration pattern than primary tumors, which is difficult to assess using conventional bread-loaf sections. Local recurrences in the head and neck region are often desmoplastic lesions characterized by high recurrence rates (6, 7). It is not uncommon for patients with this tumor type to die from complications caused by local tumor infiltration or metastases (8). Risk factors associated with recurrence, metastases and disease-specific death are as follows: Breslow thickness >2 mm, diameter >20 mm, invasion beyond subcutaneous fat, perineural involvement, poor differentiation, immunosuppression, and localization at the ear, lip and cheek (9). Recently, immunotherapy has been approved in locally advanced and metastatic cSCC. Cemiplimab (human IgG4 antibody against PD-1) was studied, and a response was observed in 13 of 26 patients (50%; 95% CI = 30 to 70). Among the patients who had a response, the duration of response exceeded 6 months in 57% (10). Cemiplimab is currently the only approved systemic immunotherapy for cutaneous SCC in Europe (2). Advanced cSCC often has a high tumor mutation burden (TMB), which may be responsive to immunotherapy, but this treatment is often contraindicated as many patients are immunosuppressed or have a history of organ transplantation (11).

ECT is an alternative treatment for cSCC that are unsuitable for standard treatments or for recurrences after standard treatments, consisting of an intravenous (IV) or intratumoral (IT) injection of a chemotherapeutic agent, such as cisplatin or bleomycin, combined with locally applied electric pulses that permeabilize tumor cell membranes to increase its cytotoxicity. In some retrospective studies and one meta-analysis, it has been reported that 20-70% of patients treated with ECT presented a good local response and disease control, while in a prospective study EURECA on cSCC patients, the rate of complete response at the 2-month follow-up was 55%, with only a 4% rate of disease progression (12–15). The results of these studies have concurred with the inclusion of ECT in the clinical guidelines of several European countries, including the UK, Italy and Germany (5, 16, 17).

The International Network for Sharing Practice in ECT (InspECT) is a pan-European collaboration of centers encompassing different specialties that treat cutaneous malignancies. The group has published analyses on outcomes for specific diseases such as cutaneous metastases from MM (18), breast cancer (19), and cutaneous tumors in the head and neck region (15, 20), as well as articles investigating specific topics of importance such as pain management (21). This database permits the collection of an extensive amount of information on large cohorts of patients, conducting more detailed analysis and investigating clinically significant variables due to data being accrued in a homogenous way.

Cohort studies on ECT in cSCC are limited to 22 patients in an exclusive paper focused on cSCC (12) and 50 patients in a study including various nonmelanoma skin cancers of the head and neck district (14). In this study, we aimed to analyze data from a larger cohort of cSCC patients included in the InspECT registry. This analysis may outline ECT benefits and disadvantages and help identify the most appropriate indications and procedural modalities.

## Patients and methods

### Database

Thirty-eight participating cancer centers across Europe prospectively entered treatment data from the start of the database from February 2008 to November 2020. Patients consented, and then data were entered into the database and subsequently updated over time. All centers uploaded data prospectively. Each institution sought approval from the Ethics Committee and data protection authority. Participation in the database was by a signed agreement. Among all included patients, a subgroup of patients with a diagnosis of cutaneous squamous cell carcinoma (cSCC) was included in the present cohort analysis. According to country-specific guidelines, patient selection was based on institutional preferences, including referral after multidisciplinary discussion for patients with symptomatic cutaneous metastasis and primary persistent/recurrent cutaneous lesions. In particular, ECT treatment was considered in (a) carefully selected patients with locally advanced disease when all other treatment options, including surgery and radiotherapy, were not feasible; (b) with persistent or recurrent primary cSCC lesions when all other treatment options, including surgery and radiotherapy, failed or were not feasible; (c) with primary naïve cSCC lesions when every other therapeutic option was contraindicated because of radically unresectable disease, at high risk of functional organ damage or because of a precarious physical condition of the patient due to comorbidities; and (d) when the patient, after being exhaustively informed, refused any other treatment option. This is an explorative article assessing treatment response as such intention to treat analysis was not performed, and only patients who were followed up for at least 45 days were included in response to treatment analysis. Reasons for short follow-up included compassionate treatment for patients whose long-term follow-up was not planned. Overall, 207 patients were selected. Of these, 162 (78%) had a sufficiently long follow-up visit to be evaluated for response to ECT treatment (i.e., 45 days) and were considered in the statistical analysis. Patients were evaluated for response to treatment after a minimum follow-up of 45 days and within a follow-up time of 90 days.

### ECT treatment

ECT was delivered based on updated European Standard Operating Procedures for Electrochemotherapy guidelines (22). Each center was responsible for the clinical decisions around the individual patient, including patient preferences (e.g., for general or local anesthesia). Bleomycin was administered either intratumorally at a dose of 1000 IU mL/cm^3^ or intravenously at 15000 IU/m^2^, and electroporation was performed using the Cliniporator^®^ (IGEA, Carpi), delivering 8 pulses of 100 ms at 1 kV/cm (voltage to electrode distance, i.e., with a 4 mm electrode gap, 400 V was delivered). Electrode choice was guided by the standard operating procedure (22).

### Response evaluation

Local response was evaluated in accordance with the modified Response Evaluation Criteria in Solid Tumors (RECIST) criteria (23) after 45-90 days of follow-up. In cases of doubt, biopsies and/or radiological examinations were performed to confirm complete response. In the case of partial response, further ECT sessions were considered. In the case of stable or progressive disease, other palliative treatment options were considered.

### Statistical analysis

Continuous variables were described by the median value and range, and categorical variables were described by the absolute number and percentage. Analysis of response to treatment has been reported as absolute number and percentage. The objective response per covariate was reported as a percentage and relative risk, together with the 95% confidence interval and Wald p value obtained by univariate logistic analysis. Statistically significant variables in the evaluation of objective response in the univariate model were taken together in a multivariate logistic model, and independent relative risk, 95% confidence interval and Wald p value were reported for each covariate included.

Local tumor control was expressed as local progression-free survival, which was the time from ECT until the date of relapse or progression or last follow-up (whichever came first). Survival curves were calculated by the Kaplan–Meier method. One-year local progression-free survival and 95% confidence interval (C.I. 95%) were calculated using Kaplan–Meier survival analysis. Comparisons between two different survival curves were made by the Mantel–Haenszel probability level. Statistical analyses were performed with NCSS 9 software (NCSS, LLC. Kaysville, Utah, USA). A p value less than 0.05 was considered statistically significant.

## Results

### Characteristics of the patient cohort

One hundred and sixty-two patients (111 males and 51 females) were included in the cohort. The median age was 80 years (range, 41-104 years). **Table 1** shows the clinical characteristics of the tumors and the previous treatments performed. Most of the patients (70%) had been pretreated as follows: 43.4% with a single treatment and 26.6% with more than one treatment. In 30% of cases, the patients were not pretreated and underwent ECT for the following reasons: the impossibility of obtaining a radical resection of the tumors; contraindication to surgery and radiotherapy because of the high risk of disfiguring and/or organ damage, or because of important comorbidities. In some other cases, the patients refused surgery and/or radiotherapy. Usually, palliative care for symptom relief was offered to these patients, trying to maintain an acceptable quality of life for as long as possible.

**Table 1:** Characteristics of the cohort of patients, nodules and treatment characteristics.

Data of patients (n = 162)				Data of nodules (n = 342)			
		N	%			N	%
Sex	Male Female	111 51	69% 31%	Lesions’ site	Head/neck/scalp Trunk Limbs	223 31 88	65% 9% 26%
ECOG	Fully active Restricted in physically strenuous activity Ambulatory and capable of all self-care Capable of only limited self-care Completely disabled Unknown	80 54 10 11 3 4	49% 34% 6% 7% 2% 2%	Current intensity delivered	0-3 Å 3-7 >7 Å	144 172 26	42% 50% 8%
Tumor presentation	Primary (persistent/recurrent*) Locally advanced	104 58	64% 36%	Electrode	Hexagonal Linear/Finger Plate Multiple	205 110 18 9	60% 32% 5% 3%
T	1 2 3 4 x	82 44 16 10 10	51% 27% 10% 6% 6%	Preirradiated lesions	Yes	59	17%
N	0 1 2 X	132 9 10 11	81% 6% 6% 7%	Lymphoedema	Yes	27	8%
M	0 1 x	151 3 8	93% 2% 5%	Lesions’ size	<30 mm ≥30 mm	241 101	70% 30%
Previous treatments	No Surgery Radiotherapy Chemotherapy Cryotherapy Electrochemotherapy Photodynamic therapy Surg+RT CT+RT Surg+CT Surg+Cryo Surg+ECT Surg+CT+RT Surg+RT+PDT Surg+RT+Cryo CT+RT+TT Surg+CT+RT+Immuno unknown	49 43 4 1 8 1 1 24 2 3 3 1 7 1 1 1 1 11	30% 27% 2% 0.6% 5% 0.6% 0.6% 15% 0.6% 2% 2% 0.6% 4% 0.6% 0.6% 0.6% 0.6% 7%				
Drug administration	Intratumoural Intravenous	28 134	17% 83%				
Anesthesia	Local/regional general	69 93	43% 57%				

Surg, surgery; RT, radiotherapy; Cryo, cryotherapy; ECT, electrochemotherapy; CT, chemotherapy; PDT, photodynamic therapy; TT, target therapy; Immuno, immunotherapy; *persistent, not responding to previous conventional treatments; recurrent, responded to previous conventional treatments but then recurred.

A median number of 1 nodule [range 1-7] was treated per ECT treatment, the median size of lesions was 21 mm [range 5-250 mm], and a median number of 15 electric pulse applications [range 1-146] were delivered per treatment. ECT was repeated in 16 patients (15 for a second time, 1 for a third time) with a partial response or a recurrence. **Table 1** shows the treatment characteristics.

### Side effects

Twenty-three patients (11%) had mostly mild (grade 1 or 2) postinterventional side effects. Of these, 9 (4%) patients had severe side effects with local ulceration and suppuration. Other indirect and direct side effects consisted of necrosis in 6 (3%), erythema in 6 (3%), odor in 4 patients (2%), flu in 3 patients (1.5%), and nausea in 2 (1%). Single cases of bleeding, itchiness, fibrosis and atrophy were observed. Grade 4 or 5 adverse effects were not observed, nor were there any patients that dropped out.

### Response

Patients were evaluated for response to treatment after a minimum follow-up of 45 days and within a follow-up time of 90 days. Responses were evaluated for patient response and lesion response.

**Figure 1:**
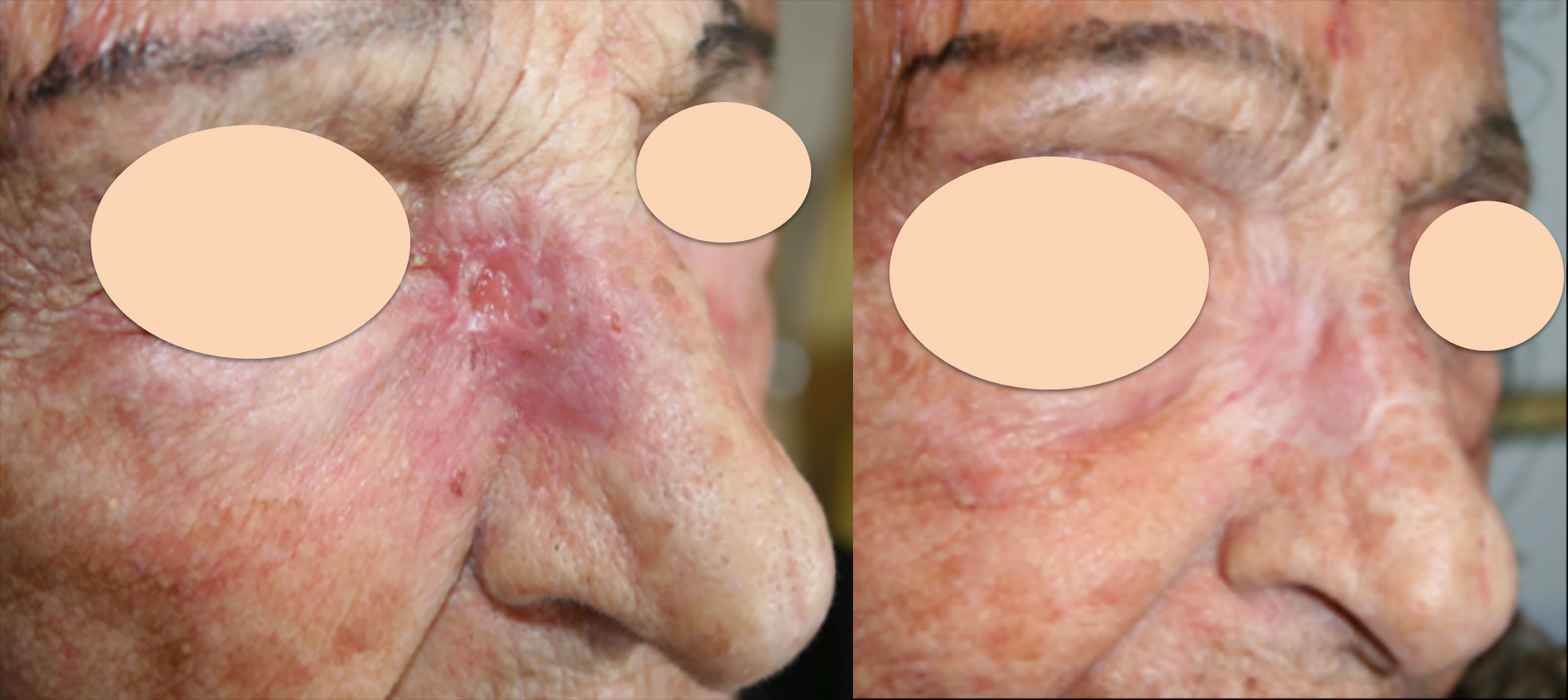
Recurrent cutaneous SCC of the inner cantus after previous surgery. Left: before ECT; Right: complete response 5 months after treatment.

Responses by patient were evaluated in 162 patients, while responses by lesions were evaluated in 342 lesions. Complete response (CR) was obtained in 61% of lesions, partial response (PR) in 18%, stable disease (SD) in 13% and progressive disease (PD) in 7% (not evaluable 1%). The response per patient was CR in 62%, PR in 21%, SD in 11% and PD in 5% (not evaluable 1%). **Figure 1** shows an example of a cSCC lesion of the inner cantus before ECT reaching and remaining in CR 5 months after treatment.

A large number of patients allowed the univariate and multivariate analysis of factors affecting the response to ECT treatment, evaluated in terms of objective response (OR=CR+PR). Univariate and multivariate analyses allowed us to evaluate the clinical and technical factors affecting the objective response to treatment and calculate the ORs% and the relative risks (RRs) for each value of the covariates. **Table 2** reports the results of the analysis with their relative significance. In the univariate model, early stage of disease (T1-T2 vs. T3-T4), low number of previous treatments, intravenous drug administration, absence of previous irradiation and small size (<3 cm) were significant predictive factors of OR. When combined in the multivariate model, only a low number of previous treatments, intravenous drug administration and small size showed a significant association with a positive outcome. A high number of previous treatments indicated long-lasting or aggressive disease that was difficult to successfully treat.

**Table 2:** Factors affecting the overall response rate: univariate and multivariate analyses.

			Univariate			Multivariate		
Variable		ORR%	RR	C.I. 95%	P value	RR	C.I. 95%	P value
Age	<80 yrs >80 yrs	78% 80%	0.91	0.54-1.57	0.7519			
Stage	T1-T2 T3-T4	82% 70%	1.95	1.02-3.72	0.0425	1.02	0.47-2.19	0.9597
# Previous tr	0 1 2 >2	86% 82% 76% 57%	1.52°	1.16-1.99	0.0057	1.52	1.09-2.12	0.0129
ECOG	0-1^+^ 2-3-4	81% 69%	1.87	0.99-3.52	0.0529			
Presentation	Primary Locally advanced	85% 75%	1.88	1.09-3.23	0.0226	1.61	0.87-3.00	0.1319
Site	Head/neck/scalp Trunk Limbs	78% 84% 83%	1.17* 1.10*	0.59-2.29 0.66-1.81	0.6455 0.7195			
Current delivered	0-3 Å 3-7 Å >7 Å	80% 81% 79%	1.08°	0.87-1.36	0.4859			
Drug administration	Intravenous Intratumoural	85% 53%	5.00	2.73-9.15	0.0001	4.83	2.41-9.67	0.0001
Preirradiated	No Yes	82% 70%	1.95	1.04-3.66	0.0319	1.06	0.49-2.29	0.8885
Lymphoedema	No Yes	79% 96%	0.15	0.02-1.09	0.0681			
Size	<30 mm ≥30 mm	83% 71%	1.96	1.14-3.39	0.0154	2.28	1.20-4.33	0.0114
Electrode	Hexagonal Other	82% 76%	1.44	0.85-2.45	0.1762			

^+^ 0, fully active; 1, restricted in physically strenuous activity; 2, ambulatory and capable of all self-care; 3, capable of only limited self-care; 4, completely disabled. *RR versus site head/neck/scalp. °RR on ordinal variables. RR, relative risk; C.I. 95%, confidence interval at 95%.

### Long-term response

The median follow-up was 5.6 months (range 1.6-47.6), with a mean of 8.6 ± 8.1 months. Recurrences occurred in 16 patients (9.9%) during the follow-up period, and disease progression occurred in 36 patients (22.2%). In the overall cohort of patients, the median time to local progression was 4.8 months (range 1-33.7 months). Among patients who reached a complete response, they could maintain their free of disease status for a mean time of 9.7 ± 8.2 months. Of the 16 patients who experienced recurrence after complete response, five were retreated with ECT and obtained a complete response again. One of them was submitted to surgical treatment to remove the recurrent lesion, and the remaining 10 patients were sent to other palliative care treatments.

**Figure 2:**
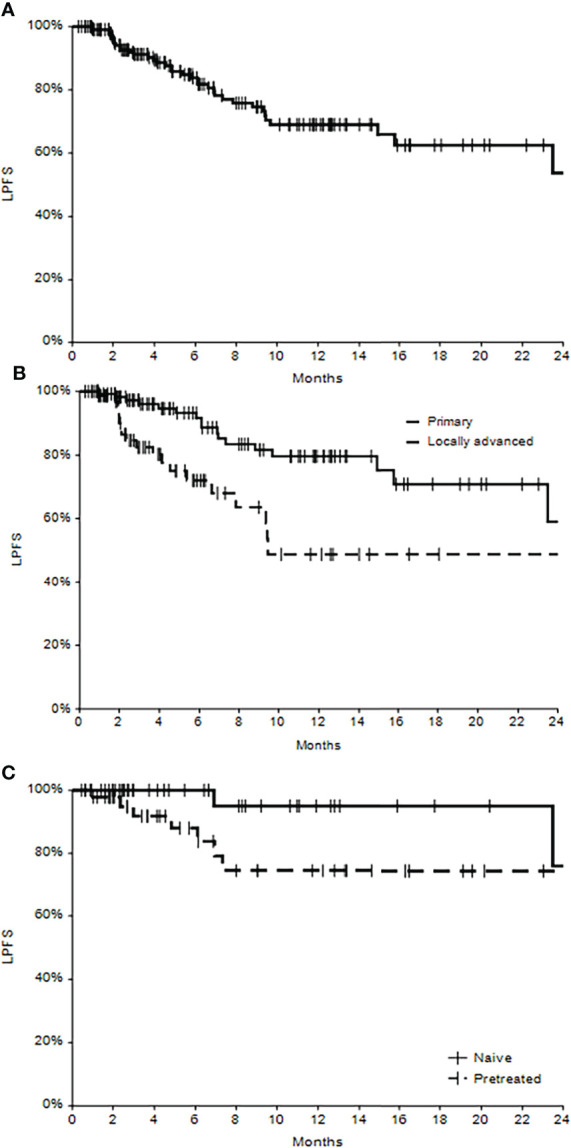
Local progression-free survival (LPFS) in the whole cohort of patients **(A)**, in subgroups of primary and locally advanced cSCC **(B)** and in subgroups of naïve and pretreated patients **(C)**.

At the last follow-up (mean 8.6 months), 78 patients had no evidence of disease (NED 48.1%), and 60 patients were alive with disease (37.0%). Death occurred in 24 patients (14.9% of 162 patients), of which 16 (66.7%) were not related to disease, and 8 (33.3%) were related to the disease. Kaplan–Meier curves show the local progression-free survival in the whole population and in primary versus locally advanced patients (**Figures 2A, B**), where the different trend was statistically significant (p=0.0016), with the survival in patients treated for primary lesions superior with respect to patients with locally advanced disease. The one-year local progression-free survival for primary patients was 80% (C.I. 95%: 70%-90%), while in locally advanced patients, it was 49% (C.I. 95%: 30%-68%) (p<0.0001).

### A subanalysis of primary tumors

A subanalysis conducted on primary lesions revealed that treatment-naïve tumors (not pretreated) showed a significantly higher percentage of complete responses (77.4%) with respect to pretreated lesions (57.7%) (p=0.0181). Even local progression-free survival seems to be higher for naïve patients, at least during the first months after ECT treatment (**Figure 2C**), even if the significance was not reached (p=0.0818). The one-year local progression-free survival in naïve patients was 95% (C.I. 95% 85%-100%), and in pretreated patients, it was 74% (C.I. 95%: 57%-92%).

## Discussion

In the present study, the patients were treated with ECT with a percentage of complete response of 62%, partial response of 21% (OR=83%) and stable, controlled disease of 11%. Side effects directly related to treatment were mild for most patients, except 9 patients with severe ulceration. Similar results were obtained by Bertino et al. (15, 24) in the EURECA study, with a 55% complete response in cSCC patients, and by Campana et al., with a 41% CR and 85% OR.

The results obtained here are of significant interest because the patients were not eligible for any other standard treatment, and they could benefit from ECT, which is an effective treatment in the majority of cases reported here.

Furthermore, the local control of the disease was relevant, as the median time to local progression was 4.8 months (range 1-33.7), with 9.9% recurrences and 22.2% disease progression. When a complete response was obtained, patients remained free of local disease for a mean time of 9.7 ± 8.2 months. Among complete responders, the duration of CR exceeded 6 months in 88% of patients. In a similar cohort of patients with locally advanced or metastatic cSCC, the safety and efficacy of immunotherapy with intravenous cemiplimab (human IgG4 antibody against PD-1) were investigated. A response to systemic therapy was observed in 13 of 26 patients (50%; 95% CI = 30% to 70%). Among the patients who had a response, the duration of response exceeded 6 months in 57% (10). In 2020, Migden et al. (25) reported the clinical activity of cemiplimab in patients with locally advanced cSCC. An objective response was observed in 34 (44%; 95% CI 32-55) of 78 patients. The best overall response was ten (13%) patients with a complete response and 24 (31%) with a partial response. Comparing the adverse event profile with systemic immunotherapy and ECT, there were significantly fewer side effects after ECT. While ECT patients from the cohort study had a total of 11% adverse events and rates of up to 48% grade 3-4, severe adverse events were observed in up to 35% of the clinical studies on cemiplimab (25, 26). According to recent real-world analyses, data of up to 77% of mild adverse events and severe adverse events of up to 45% were described (27, 28). Although cemiplimab represents the only approved systemic immunotherapy for cutaneous SCC in Europe (1), the incidence of moderate and severe adverse events is not negligible.

Long-term analysis in our cohort demonstrates that at the 1 year of follow-up, 66% of patients had maintained control of their local disease, but patients with primary lesions showed the highest rate of local control with respect to patients with locally advanced disease; 80% vs. 49% (p<0.0001). This particular result indicates that treating ECT primary (recurrent/persistent) lesions was significantly more effective than treating locally advanced lesions. Bertino et al. obtained similar results (15) in their study on head and neck cSCC.

A large number of patients/lesions in this study allowed us to perform univariate and multivariate analyses of factors affecting the response to ECT treatment in cSCC for the first time. In the univariate model, early stage of disease (T1-T2 vs. T3-T4), low number of previous treatments, intravenous drug administration, absence of previous irradiation and small size (<3 cm) were significant predictive factors of OR. When combined in the multivariate model, only a low number of previous treatments, intravenous drug administration and small size showed a significant association with OR. A high number of previous treatments indicated a long-lasting or aggressive disease that was difficult to successfully treat. Other studies have shown that pretreated lesions have a lower probability of being successfully treated with ECT (18); Bertino et al. (15), with a similar analysis conducted on cutaneous lesions of the head and neck of various histology (BCC, SCC, MM), showed that naïve tumors showed increased effectiveness of ECT and that previous surgery as single treatment the least affected the outcome compared to (chemo) radiotherapy or multiple treatments. These results would agree with our observations, even if various types of tumors were considered instead of one single histological type. In our cohort, we observed an 86.2% OR in naïve patients, 82.3% in patients who underwent a single previous treatment, 75.6% in those submitted to 2 previous therapies, and 56.7% in patients who underwent more than 2 previous treatments (p=0.0129).

We observed in our study that the influence of intravenous administration of bleomycin was significant and led to a favorable outcome with respect to intratumoral administration (p<0.0001). This finding was not significant in other analyses reported in the literature (15, 22, 29, 30). A possible explanation may be due to the difference in the histotype of sSCC with respect to other diseases as follows: it can be hypothesized that the peculiarity of the anatomical characterization of cSCC tissue, due to the strongly irregular structure but high vascularization, may be in favor of the intravenous administration of bleomycin, which more homogeneously diffuses the drug in the tissue. This observation needs to be evaluated considering the low rate of patients treated with intratumoral drug administration, which was performed in only 17% of all cases, even in the small lesions. Furthermore, the percentage of preirradiated lesions in intratumorally treated patients was 25%, higher than that of intravenously treated patients (15%); prior data had inferred a trend showing that intravenous bleomycin was preferred in previously irradiated tissues, likely due to fibrotic tissue limiting the diffusion needed for successful intratumoral drug diffusion (31); further investigation on this matter is needed.

The significant influence of lesion size in the multivariate model was confirmed, as already reported in several studies (15, 19, 24, 30, 32–34) on various types of histology.

The analysis has the following limitations: these data are from a patient registry instead of clinical trials and thus represent a real-world heterogeneous mix of patients treated with differing intentions, some for purely palliative indications and others with definitive curative intent. Patient review was not standard practice in all the participating centers; nonetheless, the InspECT group was committed to improving the quality of clinical reports through regular monitoring and analyses of the database and the publication of dedicated guidelines for researchers (35). The heterogeneous number of patients provided by each center was because not all centers started to upload patients into the registry at the same time; some centers started uploading patients’ data since the beginning (2008), and the last centers entered the InspECT group in 2019 and thus only contributed to data collection a few patients.

In the present analysis, ECT showed antitumor activity and a favorable safety profile in patients with primary unresectable/recurrent/persistent, locally advanced cSCC for whom there was no widely accepted standard of care. Furthermore, in advanced tumors, a therapeutic strategy of ECT in combination with systemic treatment, such as immunotherapy, might be even more beneficial for the patient. In fact, a preliminary report from the InspECT group showed that ECT could increase the positive effect of immunotherapy in advanced and metastatic MM patients (36). This aspect should be prospectively investigated even in cSCC patients.

## Data availability statement

The raw data supporting the conclusions of this article will be made available by the authors, upon request.

## Ethics statement

The studies involving human participants were reviewed and approved by Ljubljana: National Ethics Committee of the Republic of Slovenia (102/09/14). Padova: Padua Veneto Institute of Oncology, Institutional Approval Prot. No. 006264 – 09/04/2018; Cork: Clinical Research Ethics Committee, University College Cork, Ireland ECM 4 (IIIII) 07/05/13 & ECM 4(qq) 29/03/18. The patients/participants provided their written informed consent to participate in this study.

## Author contributions

Conceptualization: GB, AG, LC, FT, and GS. Methodology, data acquisition and analysis: GB, AG, LC, CK, HS, JG, TM, JC, PQ, EK, MM, BB, GP, FB, PM, JO, FR, AO, RP-J, GM, DM, BS, VS, WS, GB, and FT. Review and editing: GB, AG, FT, and GS. All authors have read and agreed to the submitted version of the manuscript.

## Funding

This work was conducted without any direct financial support. INSPECT is an independent group of clinicians doing research in the field of electroporation and its annual meeting is financially supported by IGEA without any involvement in the contents of the meeting.

## Conflict of interest

FT is an IGEA employer.

The remaining authors declare that the research was conducted in the absence of any commercial or financial relationships that could be construed as a potential conflict of interest.

## Publisher’s note

All claims expressed in this article are solely those of the authors and do not necessarily represent those of their affiliated organizations, or those of the publisher, the editors and the reviewers. Any product that may be evaluated in this article, or claim that may be made by its manufacturer, is not guaranteed or endorsed by the publisher.
